# The need for details for object discrimination and mnemonic discrimination

**DOI:** 10.3389/fcogn.2025.1694055

**Published:** 2025-12-09

**Authors:** Justin M. Palmer, Aidan Rhodes, Lee Ryan

**Affiliations:** 1 Department of Psychology, The University of Arizona, Tucson, AZ, United States; 2 Arizona Alzheimer's Consortium, Phoenix, AZ, United States; 3 Evelyn F. McKnight Brain Institute, Tucson, AZ, United States

**Keywords:** object recognition, mnemonic discrimination, pattern separation, aging, perirhinal

## Abstract

Both mnemonic discrimination and object recognition tasks rely on the utilization of subtle visual details. Successful mnemonic discrimination utilizes the subtle details of an event to orthogonalize highly similar episodic memories, whereas object discrimination is a memory-free skill that utilizes details in order to distinguish between similar stimuli simultaneously presented. Additionally, neuroimaging studies have implicated areas of the medial temporal lobe as important for both mnemonic discrimination and object discrimination, further suggesting that performance on these two tasks are related. However, relatively limited data assessing the relationship between these two processes exist in the literature. Seventy-one cognitively normal participants completed both the Mnemonic Similarity Task (MST) and an object discrimination task used previously by our lab. The MST displayed common objects on a white background and participants identified if the object presented was old, similar, or new compared to objects previously seen in the task. For the object discrimination task, participants were shown a pair of stimuli (blobs or squares) that varied in difficulty (hard or easy). Outcome measures included correct responses to similar objects on the MST (mnemonic discrimination) and proportion of correct matches for hard and easy blobs and squares (object discrimination). Mnemonic discrimination for similar objects were correlated only with hard blobs after correcting for age, sex, and performance on easy trials (*r* = 0.24). These results might suggest that difficulty with effectively using the subtle details to discriminate between similar objects likely have downstream consequences on mnemonic discrimination that also require the integration of multiple visual details.

## Introduction

Pattern separation relies on the ability to effectively use the subtle details of highly similar stimuli to orthogonalize them into distinct, non-overlapping representations in the brain (Yassa and Stark, 2011). The hippocampus, particularly the CA3/DG region, has been well established as a primary area involved in pattern separation in animal models (Vazdarjanova and Guzowski, 2004; Lee and Kesner, 2004) and in humans from fMRI studies (Bakker et al., 2008, 2012; Kirwan and Stark, 2007; Yassa et al., 2011a; Stevenson et al., 2020). Performance on mnemonic discrimination tasks, such as the Mnemonic Similarity Task (MST; Stark et al., 2013), is a commonly used proxy for pattern separation. The MST has been shown to reliably assess mnemonic discrimination in a wide range of populations, including cognitively normal older adults (Stark et al., 2013, 2015; Yassa et al., 2011a; Dillon et al., 2017; Holden et al., 2013; Toner et al., 2009; Stark and Stark, 2017; Doxey and Kirwan, 2015), those with depression (Déry et al., 2015, 2013; Shelton and Kirwan, 2013; Goldstein et al., 2016), anxiety (Bernstein and McNally, 2018), sleep disturbances (Doxey et al., 2018; Saletin et al., 2016), and in those with mild cognitive impairment (MCI) or Alzheimer's disease (AD; Stark et al., 2013; Yassa et al., 2010; Ally et al., 2013; Bakker et al., 2015, 2012).

Typically, the MST has an incidental encoding phase whereby participants classify whether objects are found indoors or outdoors one at a time, followed by a surprise memory phase. In this phase, participants are asked to identify if the object shown is the exact same, perceptually similar, or different from objects shown in the first phase. Accurate performance on these types of tasks has been well-documented to rely on the integrity of the DG/CA3 regions of the hippocampus based on fMRI studies (Bakker et al., 2008; Lacy et al., 2011; see also Stark et al., 2019). Models of extra-hippocampal regions that include entorhinal, perirhinal (PRC), and parahippocampal cortices are also involved as key inputs into the hippocampus (Reagh and Yassa, 2014; Burke et al., 2018). Additionally, many variations of the MST exist (Stark et al., 2019, 2023), but all have demonstrated a robust finding in the aging literature such that younger adults perform better than older adults at identifying similar objects, despite equal performance when identifying identical objects (Palmer et al., 2022; Stark et al., 2013; Holden et al., 2013; Stark et al., 2015; Toner et al., 2009; Huffman and Stark, 2017; Stark and Stark, 2017; Pidgeon and Morcom, 2014; Reagh et al., 2016). Age-related declines in mnemonic discrimination for similar objects with intact object recognition have been associated with overactivation of the CA3/DG region using fMRI (Bakker et al., 2008; Yassa et al., 2011a; Lacy et al., 2011; Yassa et al., 2011b; see also Stark et al., 2019), likely due to functional changes in the PRC (Burke et al., 2018). Burke et al. (2018) proposed a model involving two parallel pathways carrying detail and holistic information from the PRC into the hippocampus via direct and indirect projections, respectively. In aging, less activity within the PRC leads to less excitation of the detail pathway and less inhibition of the holistic pathway (Burke et al., 2018). The net result is a shift away from pattern separation and toward pattern completion, compromising someone's ability to accurately recall highly similar objects with little impact on recalling repeated objects (Yassa and Stark, 2011; Palmer et al., 2022; Burke et al., 2018; Stark et al., 2019).

Object discrimination, on the other hand, involves accurately distinguishing between similar stimuli shown simultaneously, engaging perceptual rather than mnemonic processes. Previous reviews have implicated PRC involvement with perceptual discrimination as well (Buckley and Gaffan, 2006; Murray and Bussey, 1999; Bussey et al., 2005). However, PRC involvement appears specific to differentiating stimuli on the basis of multiple features (Buckley et al., 2001; Bussey et al., 2002, 2003; Lee et al., 2005). For example, macaques with bilateral PRC ablation were able to adequately discriminate highly similar stimuli based on a single feature (i.e., size or color or shape) but were impaired when stimuli required the integration of multiple features to discriminate objects (i.e., shape and color; Buckley et al., 2001). Consistent with this, a neuroimaging study from Devlin and Price (2007) used an odd-one-out task that manipulated the type of stimulus shown (objects vs. shapes) and the difficulty (easy vs. hard). As expected, easy trials resulted in greater accuracy compared to hard trials, but no differences in accuracy between the type of stimulus was found. However, greater PRC activation was observed only for hard object trials and not hard shapes. Difficult shape trials could be differentiated on the basis of a single feature (size), whereas difficult objects relied on the ability to incorporate multiple features. Thus, greater PRC activation on the difficult object trials suggested that this region was involved with differentiating highly similar objects with multiple visual features (see also Barense et al., 2007, 2011, 2010). Age-related differences in object discrimination performance have been previously shown in animals (Burke et al., 2010, 2011) and in humans as well (Ryan et al., 2012; Gellersen et al., 2021, 2023). Ryan et al. (2012) presented older and younger adults with either a pair of blobs or squares that also varied by difficulty (i.e., degree of visual overlap). Similar to the Devlin and Price (2007), the hard squares could still be differentiated by a single feature, whereas the hard blobs had several attributes that could be different, specifically taxing the PRC to integrate all of the components in order to be successful. Older and younger adults performed equally well when distinguishing squares, regardless of the difficulty. However, when discriminating between blobs, older adults performed worse on the hard trials and no differently on the easy trials compared to younger adults. The difference between older and younger adults for hard blobs was attributed to lower anterior PRC activation using fMRI while participants completed the task.

Mnemonic discrimination and object discrimination appear to both rely on the successful utility of details, but rely on distinct and also overlapping regions of the medial temporal lobe (MTL). More specifically, mnemonic discrimination uses the details to create and store non-overlapping representations for later retrieval, which involves CA3/DG of the hippocampus. Object discrimination is a memory-free process that engages the PRC when utilizing the perceptual details to differentiate highly similar stimuli displayed simultaneously. In addition to supporting object discrimination, the PRC has been implicated as a critical region that can contribute to mnemonic discrimination performance as well (Burke et al., 2018). Therefore, it is possible that mnemonic discrimination performance could be explained by object discrimination abilities to some degree. However, relatively few studies have addressed the relationship between mnemonic discrimination and object discrimination within the same study (Gellersen et al., 2021, 2023). In one study, Gellersen et al. (2021) compared the performance of younger and cognitively normal older adults on an odd-one-out discrimination task for both objects and scenes and assessed its relationship with performance on two versions of a mnemonic discrimination task. Younger adults outperformed older adults for objects and scenes with high feature ambiguity. Additionally, perceptual discrimination significantly predicted performance on mnemonic discrimination for similar objects when the response options were forced-choice (i.e., participants were shown both the target object and the similar lure simultaneously). Perceptual discrimination did not predict memory performance when the testing format was altered to Yes/No recognition after controlling for executive function abilities among older adults.

This initial evidence supports the idea that object discrimination is an important process that is associated with performance for mnemonic discrimination for similar objects. However, the types of responses on the mnemonic discrimination task may be an important limitation to consider. A forced-choice format whereby the target and a similar lure appeared simultaneously does not completely remove perceptual discrimination demands during recall. In other words, it is unclear if a participant's performance is measuring mnemonic discrimination or re-assessing perceptual discrimination since objects are still presented simultaneously during the recall phase. On the other hand, perceptual discrimination did not explain performance when altering the format to Yes/No responses when perceptual discrimination demands were reduced. Performance on mnemonic discrimination tasks with Yes/No responses previously were shown to be a poor correlate to performance on tasks that use traditional, old, similar, and new response choices, suggesting that the mnemonic discrimination scores used may not be the optimal of proxy of pattern separation (Stark et al., 2023). Additionally, it appears that only the older adults were used in their analyses that assessed the relationship between object discrimination and mnemonic discrimination, which does not clarify how these relationships extend to younger and middle-aged adults.

This current study builds on previous work by evaluating the associations between object discrimination using blobs and squares that vary in level of difficulty (Ryan et al., 2012) and performance on a well-established three-response choice (Old/Similar/New) MST (Stark et al., 2013) among adults across the lifespan. Additionally, because these processes both decline in aging, it may be that associations are only observable in older ages (see Gellersen et al., 2021). Thus, we also investigated these relationships among older and younger adults separately as well.

## Methods

### Participants

Seventy-one cognitively normal adults from a pre-existing dataset who completed both the MST (Stark et al., 2013) and an object discrimination task (Ryan et al., 2012) were used for analyses. Participants were screened by phone for health and demographic information and excluded if they indicated any history of severe psychiatric disorders or neurological disorders, including dementia. All participants provided written and informed consent with approval by the University of Arizona's IRB. The demographics of the participants are displayed in Table 1, with an average age of 56.90, with most earning a bachelor's degree. Participants included both middle-aged and older adults (ages 30–79 years). The majority of participants were female (*n* = 54).

**Table 1:** Average age, education, and sex of participants (*n* = 71).

	**Mean age (SD)**	**Mean education (SD)**	**Sex (F : M)**
Participants (*n* = 71)	56.90	16.55	54 : 17

### Procedures

#### Mnemonic similarity task (MST)

**Figure 1:**
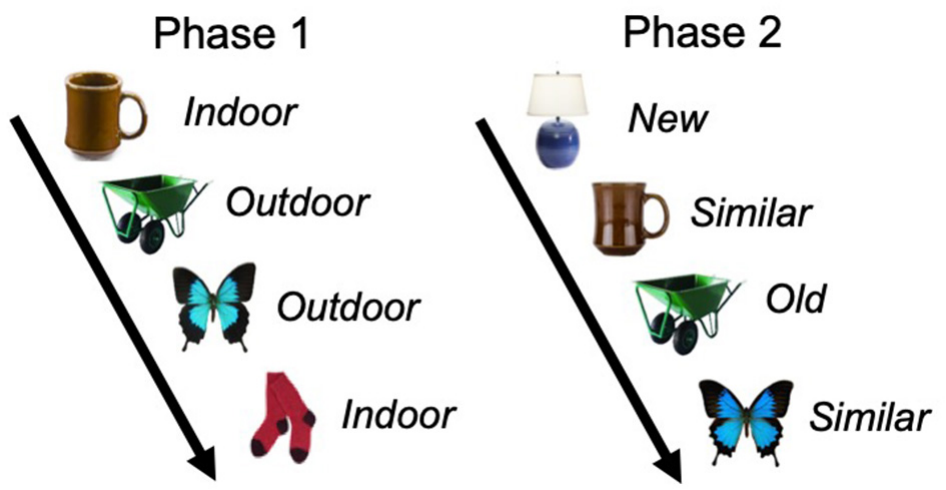
Example stimuli and response types for mnemonic discrimination. In phase 1, participants viewed objects one at a time and identified if the object is typically found indoors or outdoors. Phase 2 was a surprise memory task. Participants again viewed objects one at a time and identified if the object was old, similar, or new compared to objects seen in the first phase. The coffee mug and butterfly images are examples of similar object pairs, and the wheelbarrow is an example of old object pairs. The lamp is an example of a new object that was not previously seen in the first phase.

The version of the MST was from Stark et al. (2013), consisting of an initial incidental encoding phase where participants identified 128 common objects presented on white backgrounds on a computer as “indoor” or “outdoor” items. Immediately following this phase, participants were again shown common objects and identified them as old (presented during the encoding phase), similar (perceptually similar to stimuli from the encoding phase but not identical), or new (not presented in the encoding phase). In the recognition phase, participants were presented 192 total stimuli, 64 of which were identical to stimuli from the encoding phase, 64 of which were perceptually similar to stimuli in the encoding phase, and 64 of which were novel. In both phases, objects were displayed for 2 s, followed by a 0.5 second inter-stimulus interval. The primary outcome for mnemonic discrimination for similar objects was calculated as the proportion of similar objects correctly identified as “similar” corrected for false alarms (novel objects identified as similar), which is also known as the lure discrimination index in previous studies (see Stark et al., 2013). Object recognition was also calculated as the proportion of correctly identified old objects, corrected for false alarms. See Figure 1 for example stimuli and depiction of responses for both phases of the MST.

#### Object discrimination task

**Figure 2:**
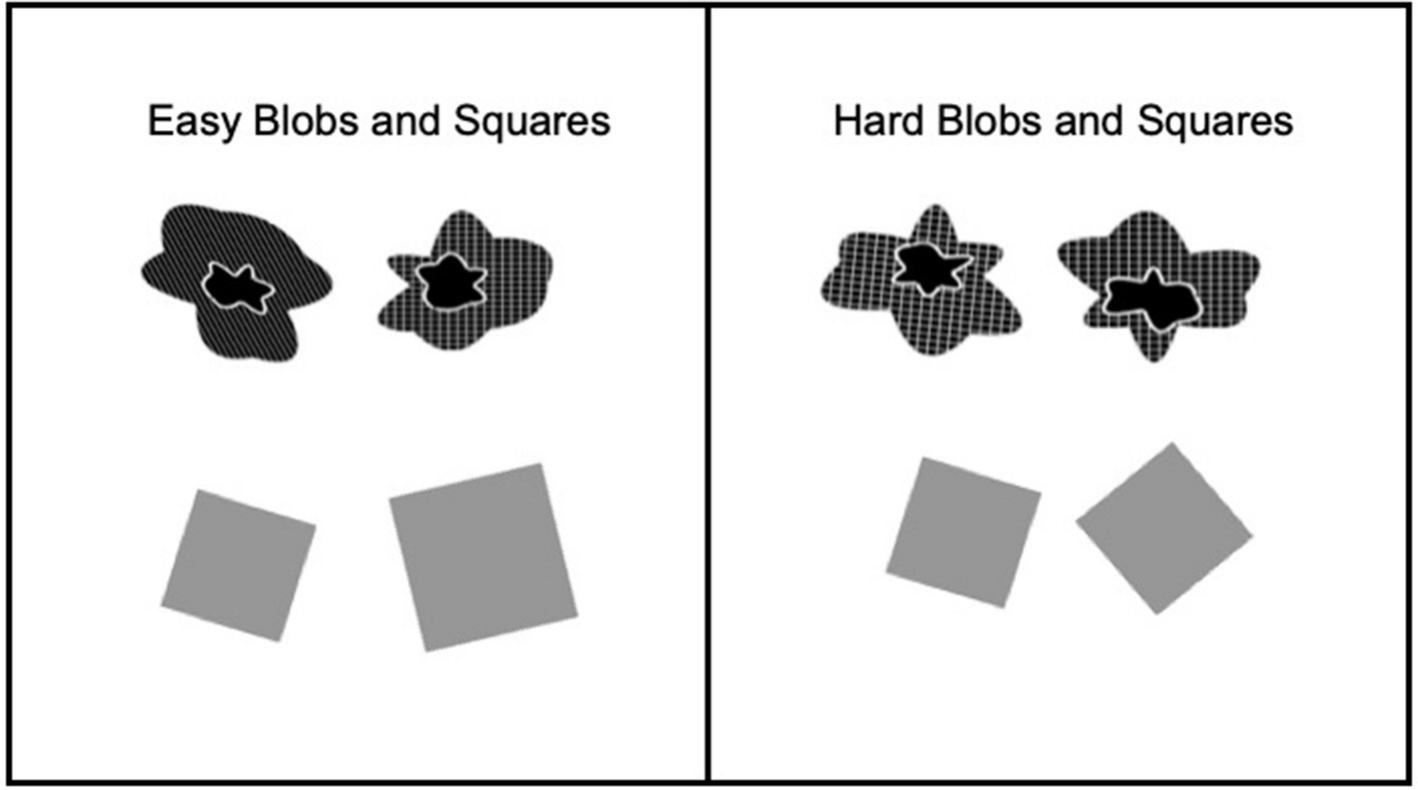
Hard and easy blobs and squares used in the object discrimination task. Stimuli were presented simultaneously, and participants responded with “match” or “non-match” to each pair.

The object discrimination task was identical to the task used by Ryan et al. (2012) and was administered on a computer using E-prime. In the task, participants were asked to identify whether two simultaneously-presented stimuli are the same or different. Stimuli are categorized as “blobs” and “squares,” and the pairs come in two difficulty levels: hard and easy. Each blob consisted of two shapes nested inside of each other with a pattern filling the space between the edge of the inner shape and the edge of the outer shape. The two shapes and the fill pattern are the three attributes in which the blobs can differ. For easy blobs trials, the blobs differ from each other on two or three of their attributes; in hard blobs trials, the blobs differ on only one attribute. The squares have only one attribute—their size. Easy squares have larger size differences (between 5 and 8 mm), and hard squares have smaller size differences (between 2 and 4 mm; Figure 2). Participants indicated that two simultaneously presented stimuli were the same by pressing the “1” key and that they were different by pressing the “2” key. Participants completed 25 practice trials with feedback followed by 200 experimental trials. The experimental trials consisted of 50 trials for each type of object (hard blobs, easy blobs, hard squares, and easy squares). Trials were randomized and presented in a fixed order. Each trial was presented for a maximum of 5 s, but the task progressed after the participant responded. Stimulus pairs were rotated when presented on the screen (consistent with Ryan et al., 2012), and participants were explicitly told about this prior to beginning the practice items. Primary dependent measures included correct identification of hard and easy blobs and squares, also corrected for false alarms. False alarms were calculated as responding “different” to matching stimuli, within each trial type.

The plan of analysis included correlations between mnemonic discrimination for similar objects and object recognition performance and all four variables from the object discrimination task: easy blobs, easy squares, hard blobs, and hard squares. We hypothesized that trials involving complex, similarity judgments among stimuli with multiple features would be related to mnemonic discrimination performance on the MST. Therefore, we predicted that only the hard blobs would be significantly correlated with mnemonic discrimination for similar objects. All data analyses were completed using IBM Statistical Program for the Social Sciences (SPSS). Power analysis for moderate correlations given the sample size (*n* = 71) at a significance level of 0.05 was 0.73.

## Results

**Figure 3:**
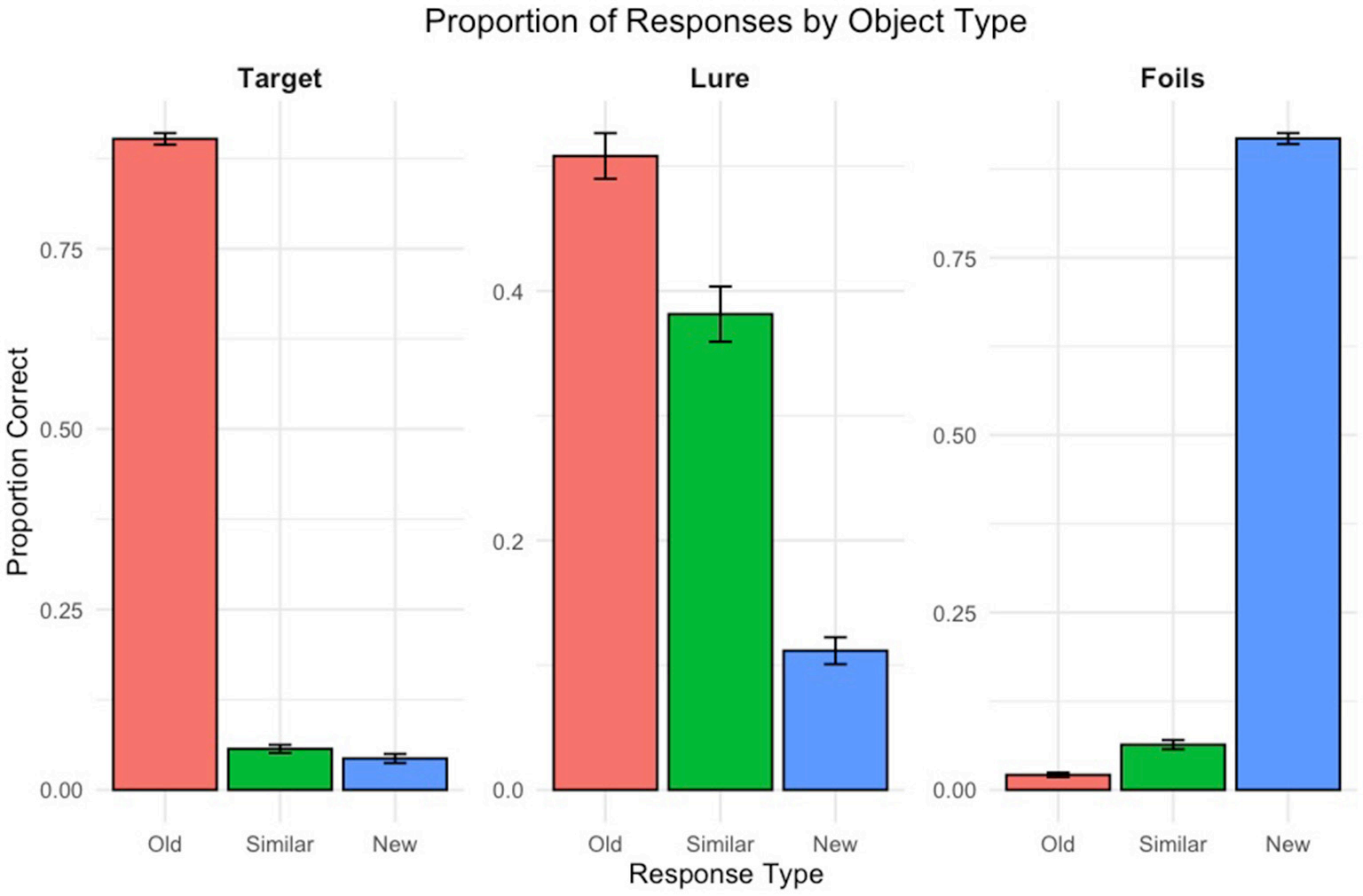
The proportion of responses (old, similar, new) for each object type (Target, Lure, Foil) for all participants. Error bars represent standard error of the mean.

The proportion of responses for each trial type on the MST is displayed in Figure 3. Primary analyses included mnemonic discrimination for similar objects and object recognition. Mnemonic discrimination performance was calculated as the proportion of correctly identified similar objects (corrected for false alarms) on the MST as a proxy for pattern separation. Object recognition was calculated as the proportion of correctly identified old objects (corrected for false alarms). On the object discrimination task, total accuracy as well as the accuracy for each trial type (easy and hard blobs and squares) were calculated. Average performance from the two tasks is displayed in Table 2, with all measures corrected for their respective false alarms. As expected, easy trials resulted in better performance than the hard trials, regardless of the stimulus type, *F*(1,70) = 87.31, *p* < 0.001, $\eta_{p}^{2}$ = 0.56. Blob trials were also more difficult compared to squares *F*(1,70) = 43.20, *p* < 0.001, $\eta_{p}^{2}$ = 0.38, presumably due to the increased demands of integrating and evaluating a stimulus with multiple features. No interaction between stimulus type and difficulty level was found, *F*(1,70) = 7.32, ns.

**Table 2:** Proportion correct (SD) for each measure on the MST and on the object discrimination task (easy/hard blobs and easy/hard squares).

**Measures**	**Proportion correct (SD)**
Mnemonic discrimination (similar objects)	0.31 (0.19)
Object recognition	0.88 (0.07)
Object discrimination (all trials)	0.78 (0.05)
Easy blobs	0.64 (0.25)
Easy squares	0.86 (0.12)
Hard blobs	0.40 (0.18)
Hard squares	0.55 (0.24)

Each measure is corrected for false alarms.

**Figure 4:**
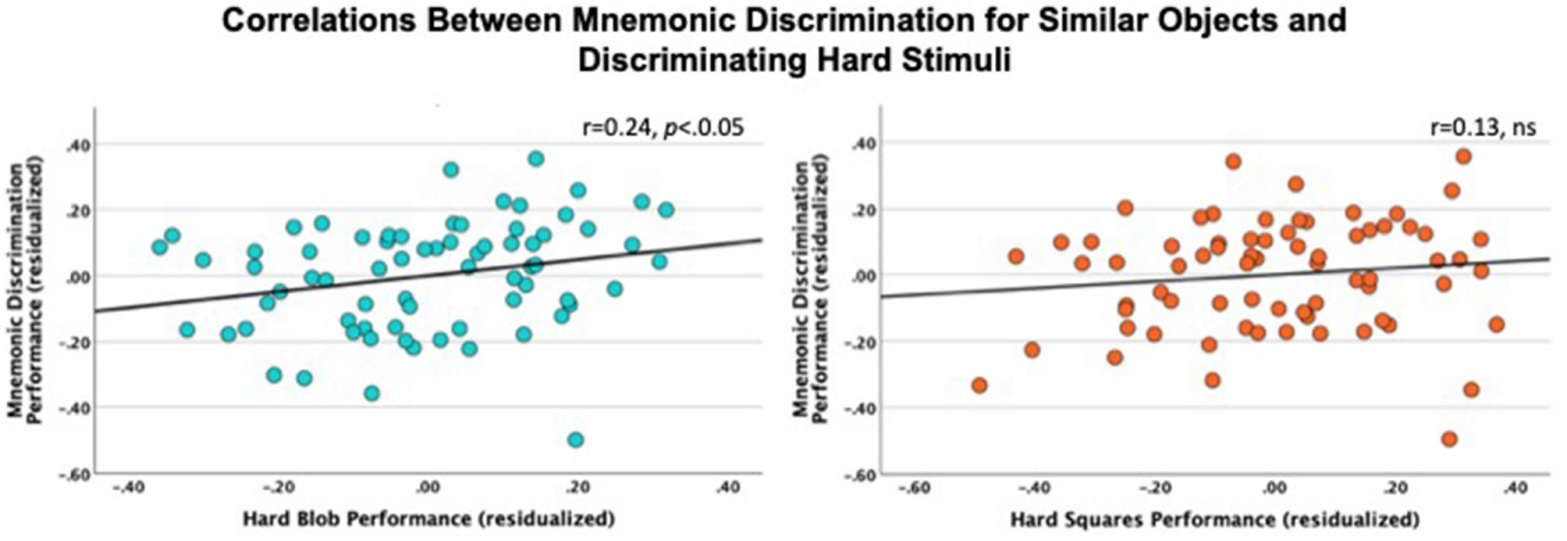
Scatter plots of the relationship between mnemonic discrimination for similar objects and discrimination of hard blobs (blue) and squares (orange) after accounting for age, sex, and performance on easy trials. Mnemonic discrimination for similar objects (y-axis) was correlated with hard blobs (*r* = 0.24, *p* < 0.05) but not hard squares (*r* = 0.13, ns) (x-axis).

Correlation analyses were conducted to understand the relationship between mnemonic discrimination for similar objects and object recognition and each of the outcome measures from the object discrimination task. Mnemonic discrimination for similar objects was significantly correlated with hit rates for both hard blobs (*r* = 0.30, *p* = 0.01) and squares (*r* = 0.32, *p* < 0.01). Importantly, mnemonic discrimination for similar objects was not correlated with the easy trials, blobs (*r* = −0.22, ns) or squares (*r* = 0.01, ns). Object recognition was only correlated with hard blobs (*r* = 0.26, *p* < 0.05). As an alternative approach to control for age and also considers a hierarchical-representational view (Kent et al., 2016), partial correlations were conducted to control for age, sex, and accuracy on easy trials. Mnemonic discrimination for similar objects was correlated with hard blobs, even after controlling for age, sex, and easy blobs trials (*r* = 0.24, *p* < 0.05) but was not correlated with hard squares after controlling for age, sex, and easy squares (*r* = 0.13, ns; Figure 4). Participants appeared to have a higher proportion of responding old to similar objects relative to responding similar (a pattern that tends to reflect a bias toward pattern completion). Therefore, we calculated a pattern separation efficiency index (PSEI) as a more precise metric of pattern separation in a *post-hoc* analysis (García-Rueda et al., 2024; Toner et al., 2009). PSEI was calculated by subtracting old responses to similar objects from similar responses to similar objects. Overall results did not change using this metric, as PSEI was correlated with hard blobs (*r* = 0.30, *p* < 0.05) but not hard squares (*r* = 0.06, ns) after controlling for age, sex, and easy trials. Additionally, corrected recognition was no longer correlated with hard blobs after controlling for age, sex, and easy blob accuracy (*r* = 0.17, ns). We interpret these findings to suggest that the observed relationships are not likely to be driven by age, sex, or general ability primarily, but rather reflect shared demands between mnemonic discrimination and object discrimination when stimuli contain multiple, complex features.

Primary analyses included all participants, regardless of age. Nonetheless, the robust literature of age-related declines in both object discrimination and mnemonic discrimination might suggest that the relationships for these cognitive processes may be particularly evident in older ages. When we grouped participants equal to or above the age of 65 (*n* = 28) and below 65 (*n* = 43), the correlations between mnemonic discrimination for similar objects and hard blobs/squares did not persist in either group, though the correlation between mnemonic discrimination for similar objects and hard blobs was marginal among older adults (*r* = 0.37, *p* = 0.051).

## Discussion

The major finding in this study was that correctly identified similar objects was moderately related to the ability to discriminate between highly similar squares and blobs. However, after accounting for age, sex, and performance when discriminating between stimuli with less feature complexity, identifying similar objects was only related to the discrimination of hard blobs. *Post-hoc* analyses using an efficiency index (PSEI) that accounted for old responses to similar objects also demonstrated consistent results. Analyzing the relationship between mnemonic discrimination and visual discrimination using this metric controls for a bias toward pattern completion, a shift that tends to occur in aging. Therefore, our results highlight a specific relationship between mnemonic discrimination for similar objects and accurately distinguishing between stimuli with multiple visual features, regardless of age.

Similar findings have been previously shown among older adults as a function of task design, whereby perceptual discrimination of highly overlapping objects and scenes explained mnemonic discrimination performance only during forced-choice recognition (Gellersen et al., 2021). However, forced-choice responses where the target and lure stimuli are presented simultaneously does not completely remove perceptual discrimination ability on memory recall in this paradigm. This relationship was attenuated when objects were no longer presented simultaneously by using Yes/No response choices. However, this may not be the optimal format to assess mnemonic discrimination, which could explain the null findings. Our data demonstrated that when visual discrimination is better accounted for by using the traditional three-response choices (Old/Similar/New), mnemonic discrimination for similar objects relates to object discrimination with complex features only. Additionally, the previous study only included older adults in their analyses of perceptual discrimination and mnemonic discrimination, despite younger adults being enrolled in the overall study. Therefore, the observed associations between mnemonic discrimination and perceptual discrimination among younger/middle-aged adults extend this previous research.

The significant relationship between object discrimination and mnemonic discrimination for similar objects would be consistent with the emerging research that suggests pattern separation is associated with additional cognitive processes beyond memory (Chwiesko et al., 2023; Kent et al., 2016). The role of executive functions is an example of this, as previous studies have implicated executive functions as particularly important for mnemonic discrimination. A study of younger and older adults found a significant relationship between executive functioning and episodic memory with performance on the MST. Moreover, their dominance analysis indicated that executive functioning was the strongest predictor for mnemonic discrimination (Jensen et al., 2023). Additionally, executive functioning composite scores including measures of working memory, switching, and verbal fluency predicted performance on a Yes/No format of mnemonic discrimination among older adults (Gellersen et al., 2021). Consistent with this, our lab has recently found a significant relationship between pattern separation using the MST and executive function using the classic Flanker task to assess inhibition among younger and older adults (Palmer et al., under review). Within neuroimaging studies, connectivity analysis from an fMRI study identified the dorsomedial prefrontal cortex (dmPFC) in addition to the hippocampus and parahippocampus as key regions involved when completing a continuous version of the MST among older adults (Nash et al., 2021). The authors concluded that the dmPFC would be involved with executive functioning processes such as cognitive control when completing these types of tasks, further suggesting that other cognitive domains beyond memory are critical for accurate mnemonic discrimination. The results in the present study suggest that object discrimination may be another critical process that relates to mnemonic discrimination as well. More specifically, object discrimination may be an important first step that relates to downstream mnemonic discrimination ability, though correlation analyses limit the interpretation of the directionality of the effect.

Although the association among older adults alone was marginal in the current study, the contribution of perceptual discrimination on mnemonic discrimination may have particular relevance for older adults. Younger adults have consistently been shown to perform better than older adults on object discrimination tasks (Ryan et al., 2012) and on mnemonic discrimination tasks with a range of visually-based stimuli, including faces (Kirwan and Stark, 2007), scenes (Stark and Stark, 2017), words (Ly et al., 2013), and spatial locations (Stark et al., 2010; Reagh and Yassa, 2014; Holden et al., 2012; Reagh et al., 2014, 2016). If distinguishing between details is more difficult for older adults at a perceptual level, it follows that creating a unique representation retaining those subtle details would also be difficult for them. Consistent with these behavioral findings, age-related functional changes to the PRC appear to have implications for both processes (Burke et al., 2018). Age-related reductions in PRC activation can lead to declines in detailed information being delivered into the hippocampus for effective pattern separation (Burke et al., 2018). Furthermore, lower PRC activation among older adults was related to greater difficulty when discrimination required the integration of multiple, detailed features rather than a single feature, such as the size of a stimulus. Taken together, age-related changes in PRC functioning may negatively impact object discrimination and, therefore, compromise the information being projected into the hippocampus for successful pattern separation. In other words, the quality of the information being sent by the PRC would likely have implications for someone's ability to adequately orthogonalize highly similar memories. Thus, older adults would be particularly vulnerable to poorer performance on both tasks compared to younger adults who do not typically show difficulty with object discrimination. However, when participants were grouped by age in the current study, the correlations in the present study were slightly attenuated. Likely the lower number of older adults (*n* = 28) limited the ability to detect age-specific relationships. It should be noted that the attenuation of correlations after splitting the age groups does not directly compare the changes in the relationship, and future studies with larger samples would be in a better position to directly test this relationship (Diedenhofen and Musch, 2015).

The relationship between mnemonic discrimination for similar objects and hard squares did not persist when controlling for accuracy of distinguishing between easy squares. The dissociation between hard blobs and hard squares suggests that our findings are not likely a function of difficulty, as the correlation between hard blobs and mnemonic discrimination would have likely been attenuated as well after correcting for easy trials. Discriminating between hard blobs and hard squares both rely on the need to evaluate the subtle perceptual details. However, the hard blobs require the integration of multiple features, whereas discriminating between the squares only requires the differentiation of one feature, size. Mnemonic discrimination for everyday objects would also require the evaluation and integration of multiple visual features. In other words, the associations found with the hard blobs and mnemonic discrimination is not simply because both tasks used highly similar stimuli, but rather it may be because both tasks share the need to integrate multiple visual features and utilize subtle details to be successful. As described above, this specific finding would be consistent with the shared functioning of the PRC as well. Future fMRI studies would be a critical next step to assess PRC activation when completing both tasks within the same study.

## Conclusions

The relationship between object discrimination and mnemonic discrimination both rely on the ability to utilize perceptual details. Although the directionality of the relationship cannot be determined with correlations, potentially the ability to accurately distinguish between two similar objects with multiple features has direct implications on downstream orthogonalization for later retrieval. Additional factors such as visual acuity may also be an important consideration as an even further upstream process (Davidson et al., 2019). We were not able to objectively measure visual acuity in the current study, though the relationship between visual acuity and mnemonic discrimination does not appear to persist in studies with larger samples (Jensen et al., 2023). For detailed information of perceptual stimuli to be encoded into long-term memory separately from another highly similar stimulus, the representation of the object must be accurately differentiated. Perceptual discrimination ability may be a critical variable to account for when assessing mnemonic discrimination ability in future studies.

## Data Availability

The raw data supporting the conclusions of this article will be made available by the authors, without undue reservation.
